# Improved long-term cardiovascular outcomes after intensive versus standard screening of diabetic complications: an observational study

**DOI:** 10.1186/s12933-019-0922-1

**Published:** 2019-09-16

**Authors:** Mario Luca Morieri, Enrico Longato, Marta Mazzucato, Barbara Di Camillo, Arianna Cocchiglia, Lorenzo Gubian, Giovanni Sparacino, Angelo Avogaro, Gian Paolo Fadini, Saula Vigili de Kreutzenberg

**Affiliations:** 1 Department of Medicine, University of Padova, Via Giustiniani 2, 35128 Padua, Italy; 2 Department of Information Engineering, University of Padova, 35131 Padua, Italy; 3 Arsenàl.IT Consortium, Veneto Region, 31100 Treviso, Italy; 4 Azienda Zero, Veneto Region, 35131 Padua, Italy

## Abstract

**Background:**

Complication screening is recommended for patients with type 2 diabetes (T2D), but the optimal screening intensity and schedules are unknown. In this study, we evaluated whether intensive versus standard complication screening affects long-term cardiovascular outcomes.

**Methods:**

In this observational study, we included 368 T2D patients referred for intensive screening provided as a 1-day session of clinical–instrumental evaluation of diabetic complications, followed by dedicated counseling. From a total of 4906 patients, we selected control T2D patients who underwent standard complication screening at different visits, by 2:1 propensity score matching. The primary endpoint was the 4p-MACE, defined as cardiovascular mortality, or non-fatal myocardial infarction, stroke, or heart failure. The Cox proportional regression analyses was used to compare outcome occurrence in the two groups, adjusted for residual confounders.

**Results:**

357 patients from the intensive screening group (out of 368) were matched with 683 patients in the standard screening group. Clinical characteristics were well balanced between the two groups, except for a slightly higher prevalence of microangiopathy in the intensive group (56% vs 50%; standardized mean difference 0.11, p = 0.1). Median follow-up was 5.6 years. The adjusted incidence of 4p-MACE was significantly lower in the intensive versus standard screening group (HR 0.70; 95% CI 0.52–0.95; p = 0.02). All components of the primary endpoint had nominally lower rates in the intensive versus standard screening group, which was particularly significant for heart failure (HR 0.43; 95% CI 0.22–0.83; p = 0.01).

**Conclusion:**

Among T2D patients attending a specialist outpatient clinic, intensive complication screening is followed by better long-term cardiovascular outcomes. No significant effect was noted for cardiovascular and all-cause mortality and the benefit was mainly driven by a reduced rate of hospitalization for heart failure.

## Background

Diabetes mellitus is associated with reduced life expectancy owing to severe multi-organ complications. On average, a 50-year-old man without a history of cardiovascular disease (CVD) but with diabetes dies 6 year earlier than a subject without diabetes, with the majority of premature causes of death being attributable to CVD [1]. Despite the global decline in mortality and incidence of CVD over the last decades, seen both in subjects with and without diabetes, the latter still experience a 2–3 times higher risk as compared to non-diabetic matched controls [1–3]. This higher risk, combined with the high prevalence of subjects that already have cardiovascular complications at the time of diabetes diagnosis [4–6], makes the screening for chronic complications highly recommended for all patients with type 2 diabetes (T2D). Early identification of complications can result in the prescription of more aggressive treatment regimen that have the potential to slow their natural progression. For example, a diagnosis of autonomic neuropathy by screening identifies patients at risk for adverse cardiovascular outcomes [7, 8]. Thus, results of complication screening can yield a better awareness of the interplay among micro- and microangiopathies [9, 10], and help clinicians to tailor treatment and the outpatient schedule visits [11]. In general, evidence of absence of complications or presence of multiple severe complications can both have important implications for appropriate management of diabetes and comorbidities [12]. Poor achievement of glucose targets [13] and the global under-utilization of glucose lowering drugs provided with cardiovascular protective effects [14, 15] suggests that a better screening of cardiorenal complications can promote improvements in therapeutic appropriateness for T2D.

However, the intensity and schedules of complication screening can vary considerably according to the healthcare setting (e.g. primary care versus specialist care) and availability of resources. Diagnostic workup for chronic diabetic complications should not be provided on demand based on patient’s complains, but based on routine schedules as recommended by evidence-based international standards [16].

There is mounting evidence of lower overall mortality in diabetic patients treated by a multi-disciplinary team within diabetes specialist outpatient services versus treatment in primary care [17]. However, it is still unknown whether, even within the same system of health care provision, screening intensity affects cardiovascular outcomes. We hypothesize that providing patients with an intensive session of multi-disciplinary screening of complications could result in better outcomes than when complications are screened in staggered sessions. Thus, the aim of this study was to evaluate whether an intensive screening of chronic complications resulted in long-term improvement of cardiovascular outcomes as compared to standard screening.

## Methods

### Study design

This was a single-center, retrospective, observational study. Clinical data for subjects attending the Diabetes Outpatient Clinic of the University Hospital of Padova between January 2007 and December 2015 were collected from structured electronic medical records (EMR) linked with hospital discharge codes and causes of death, with outcome data collected up to November 2018.

### Study population

During the study period, 12,930 patients attended the clinic at least once, 6701 of whom had a diagnosis of T2D, with 6469 subjects successfully and anonymously linked with administrative data, including hospital discharge codes and causes of death. 5274 subjects with complete baseline data available were included in the analyses, 368 of whom were exposed to the intensive screening visit. A Diagram flow chart for selection of patients is shown in Additional file 1: Figure S1.

For patients included in the intensive screening group, the index date was set as the date patients accessed the clinic for the screening workup. For patients in the control group, since multiple visits were available in the period considered in this study, each visit with complete data was considered as a usable index date. For those subjects in the control group with more than one visit per year, the index date used in the matching procedure was randomly selected among the visits available in that year. Similarly, in the multivariable analyses, for each patient in the control group, the index visit was randomly selected among available visits.

The following baseline data were extracted from EMR at the time of the index date: age, sex, type 2 diabetes diagnosis, diabetes duration, glucose lowering medications and other medications, HbA1c, body mass index (BMI), systolic and diastolic blood pressure, total cholesterol, HDL cholesterol, triglycerides and LDL cholesterol (estimated with the Friedewald formula [18]), serum creatinine, estimated glomerular filtration rate (eGFR, calculated according to the CKD-Epidemiology Collaboration equation [19]), presence of micro- or macroalbuminuria, diagnosis of diabetic retinopathy, diagnosis of diabetic neuropathy, history of coronary artery disease (CAD, defined as a history of myocardial infarction, angina, revascularization or evidence of significant CAD at coronary angiography), peripheral arterial disease (PAD, defined as claudication or rest pain with evidence of leg artery stenosis, or ankle-brachial index < 0.9, or revascularization), and cerebrovascular disease (CerVD, defined as a past history of stroke or transient ischemic attack, or carotid artery revascularization). Macroangiopathy was defined as the presence of CAD, PAD, CerVD or carotid artery plaques, whereas microangiopathy was defined as the presence of retinopathy, neuropathy, micro/macro-albuminuria or chronic kidney disease (CKD) stage III or higher (defined as a eGFR < 60 ml/min/1.73 m^2^). Whenever baseline data were not available at the index date, these were retrieved from the closest information available in prior visits up to a maximum of 365 days prior to the index date (with the exclusion of data on age, diabetes duration, BMI and medications that needed to be collected at the index date).

### Exposure and comparison

Whether patients received intensive or standard screening was decided by the treating diabetologist and was not randomized. Intensive screening for diabetic complications was provided in a 1-day session and included evaluation of risk factors, digital fundoscopy, neuropathy autonomic tests and Michigan Neuropathy Screening Instrument (MNSI), eGFR and albumin excretion rate, foot care, electrocardiogram, echocardiogram, and carotid ultrasound. At the end of the screening day, patients received extensive counseling with indications on the need for additional diagnostics, eventual therapeutic modifications, and advises on lifestyle. By means of propensity scores, patients in the intensive screening group were matched 1:2 with T2D patients attending the same clinic in the same year, who underwent routine screening for micro- and macroangiopathy at different visits during the same year (total n = 4906). After the index date, subjects of both groups were exposed to the same health care pathway with routine visits scheduled according to standard of care and physician decisions.

### Outcomes

The primary outcome was the first occurrence of 4-point major adverse cardiovascular events (4p-MACE), defined as cardiovascular mortality, non-fatal myocardial infarction, non-fatal stroke, or hospitalization for heart failure. Secondary outcomes were the 3-point MACE (4-p MACE without heart failure), individual components of primary outcome, and overall mortality.

Hospital discharge codes between 2007 and 2018 were obtained from the Veneto region registry, and used to identify non-fatal cardiovascular events, according to the presence in the first five discharge diagnosis of the following international statistical classification of diseases and related health problems ICD 9th edition codes for myocardial infarction (410, 411, 413 and 414), stroke or transient ischemic attack (431, 432, 433, 434, 435, 436), or heart failure (428). To limit the probability of reverse causality that would occur in case of an association between index visit and planned hospitalization for cardiovascular causes, any cardiovascular event occurring within 3 months after the index date was not considered in the outcome analyses.

From death certificates, a cardiovascular death was adjudicated according to the presence of the ICD 10th revision codes ranging from I00 to I99 (disease of the circulatory system) and F01 (vascular dementia), listed among the primary or concomitant causes of death. Deaths from unknown causes and not attributed to a non-cardiovascular cause were considered as cardiovascular.

### Statistical analysis

Continuous variables are reported as mean and standard deviation, whereas categorical variables are reported as percentage. Propensity score matching (PSM) was used to generate similar groups and compare cardiovascular outcomes in the two groups (intensive versus standard screening). All baseline characteristics specified above, including granularity for glucose lowering medications (diet, metformin alone, or innovative treatment, defined as DPP-4 inhibitors, GLP-1 receptor agonist, or SGLT-2 inhibitors), other medications (ACE inhibitors/angiotensin receptor blockers [ARBs], antiplatelet therapy, statins, or other lipid lowering drugs), expected to be associated with group assignment, were included in the propensity score (PS) model. Then, the PS was used to match each subject in the intensive screening visit group with up to 2 subjects attending the clinic in the same year and subjected to a standard screening regimen. Whenever a subject in the standard group had more than one visit available in the same year, one of such visits was randomly selected and used for the matching procedure. Matching was performed 1:2 ratio without replacement, according to a SAS macro [20], optimized to implement PSM within a maximum radius/caliper set to 0.25 SD of the PS. Covariates balance was assessed using standardized mean differences (SMD) between the groups: balance were achieved if SMD were < 0.1 and *p* value for differences between the two groups > 0.05, assessed by Student’s t test or Chi square, as appropriate. The Cox proportional hazard regression analysis was used to compare endpoint occurrence in the two matched groups, adjusted for residual imbalances.

In a sensitivity analysis alternative to the PS-matched comparison, we included all patients who participated in the intensive screening and all patients having standard screening during the study period. We thus performed a multivariable adjusted Cox regression analysis, entering all the covariates that were used to estimate PS.

Differences between the two groups in the changes of cardiovascular risk factors over time were tested by means of linear mixed models, including time, screening group and “time by group” interaction term in the model. The differences in medication use during follow-up were evaluated with a logistic regression model for repeated measure with generalized estimating equations. The estimated number of subjects needed to be exposed to the intensive screening program to avoid outcomes over the following 5 years were estimated as previously reported [21].

The study was conducted in accordance with the principles of the Declaration of Helsinki and approved by local institutions and ethical committees. All patients provided written informed consent. Statistical analyses were performed using SAS version 9.4 (TS1M4) and the statistical significance level was conventionally set at 0.05.

## Results

### Baseline characteristics

After PSM, 357 patients from the intensive screening group were matched with 683 patients in the standard screening group (baseline characteristics of subjects in the entire cohort of 5290 subjects are described in Additional file 1: Table S1). As shown in Table 1, only a few proportion of patients in the standard and intensive groups had a prior cardiovascular event (8.6% and 8.7%, respectively), while 42% had evidence of macroangiopathy, either symptomatic or asymptomatic. Overall, the major cardiovascular risk factors were well balanced between the two groups. However, the baseline prevalence of microangiopathy remained slightly imbalanced, being higher in the intensive screening group (55% versus 50%), but not significantly (SMD = 0.11 and p = 0.10).

**Table 1:** Baseline characteristics of study patients

Characteristics	Standard screening (N = 683)	Intensive screening (N = 357)	SMD	p
Age	60.7 ± 12.1	60.0 ± 8.9	0.07	0.29
Female (%)	238 (34.8%)	121 (33.9%)	0.02	0.76
Duration of diabetes (years)	7.9 ± 6.6	8.4 ± 7.5	− 0.08	0.27
BMI (kg/m^2^)	29.4 ± 5.0	29.2 ± 5.0	0.04	0.51
Systolic BP (mmHg)	135.7 ± 19.9	136.3 ± 16.8	− 0.03	0.64
Dyastolic BP (mmHg)	81.8 ± 11.3	81.7 ± 10.1	0.00	0.96
HbA1c (%) (mmol/mol)	7.5 ± 1.5 (58 ± 12)	7.5 ± 1.4 (58 ± 11)	− 0.03	0.61
Total cholesterol (mg/dl)	183.0 ± 40.5	183.1 ± 35.9	0.00	0.99
HDL-cholesterol (mg/dl)	49.2 ± 13.7	49.4 ± 14.6	− 0.01	0.84
Triglicerides (mg/dl)	131.8 ± 68.9	133.1 ± 75.2	− 0.02	0.78
LDL cholesterol (mg/dl)	107.2 ± 35.3	107.1 ± 31.2	0.00	0.95
Creatinine (mg/dl)	0.87 ± 0.20	0.86 ± 0.21	0.03	0.69
eGFR (ml/min/1.73 m^2^)	87.0 ± 17.7	87.9 ± 16.9	− 0.05	0.43
Albuminuria				0.83
Normoalbuminuria N (%)	495 (72.5%)	254 (71.1%)	0.03	
Microalbuminuria N (%)	155 (22.7%)	83 (23.2%)	− 0.01	
Macroalbuminuria N (%)	33 (4.8%)	20 (5.6%)	− 0.03	
Medical treatment				
Any glucose-lowering drugs N (%)	579 (84.8%)	312 (87.4%)	− 0.08	0.25
Metformin alone N (%)	240 (35.1%)	118 (33.1%)	0.04	0.50
Insulin N (%)	130 (19.0%)	76 (21.3%)	− 0.04	0.39
Innovative therapies N (%)	58 (8.5%)	35 (9.8%)	− 0.05	0.48
ACEi/ARBs N (%)	452 (66.2%)	242 (67.8%)	− 0.04	0.60
Statin N (%)	436 (63.8%)	235 (65.8%)	− 0.04	0.52
Lipid-lowering therapy N (%)	474 (69.4%)	253 (70.9%)	− 0.04	0.62
Anti-platelet therapy N (%)	224 (32.8%)	113 (31.7%)	0.03	0.71
Medical history				
CAD or CerVD events N (%)	59 (8.6%)	31 (8.7%)	0.00	0.98
Macroangiopathy N (%)	285 (41.7%)	150 (42.0%)	− 0.01	0.93
Carotid atheroma or PAD, %	37.6%	40.1%		
CAD, %	8.0%	8.1%		
CerVD, %	0.7%	0.6%		
Microangiopathy N (%)	344 (50.4%)	199 (55.7%)	− 0.11	0.10
Diabetic nephropathy, %	32.3%	31.9%		
Diabetic retinopathy, %	33.8%	30.3%		
Diabetic neuropathy, %	15.2%	19.0%		
CKD N (%)	59 (8.6%)	29 (8.1%)	0.02	0.78

*SMD* standardized mean difference, *BMI* body mass index, *eGFR* glomerular filtration rate, *CKD* chronic kidney disease, *ACEi* angiotensin converting enzyme inhibitors, *ARBs* angiotensin receptor blockers, *CAD* coronary artery disease, *CerVD* cerebrovascular disease, *PAD* peripheral arterial disease

### Cardiovascular outcomes

The median follow-up was 5.6 years (IQR 3.7–8.6) and was similar in the standard and intensive screening group (5.6, IQR 3.6–8.6 versus 5.6, IQR 3.8–8.8, respectively), follow-up time was censored at 10 years given the small numbers of subjects with longer follow-up.

**Fig. 1:**
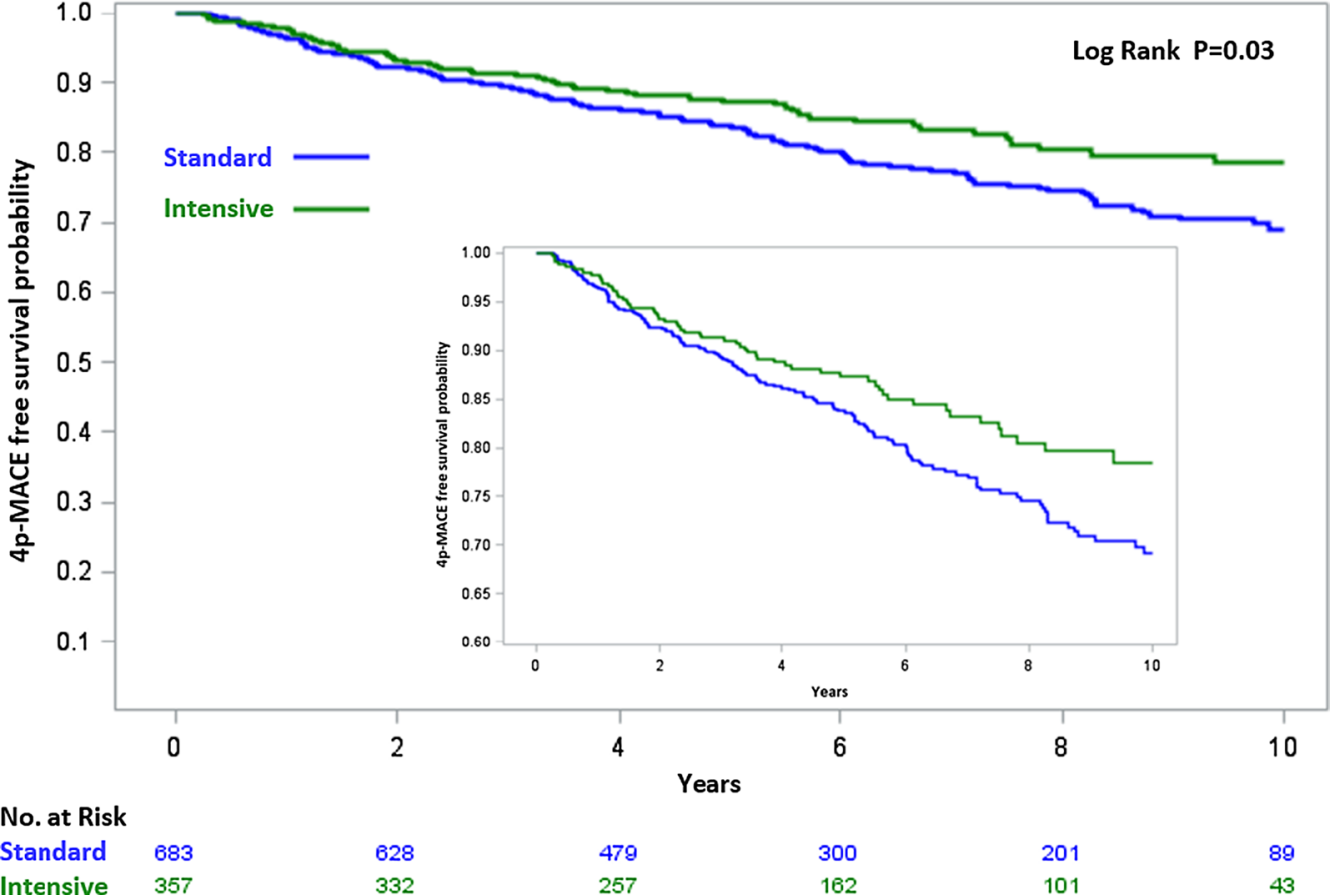
Kaplan–Meier curves for the primary endpoint in the two groups

**Fig. 2:**
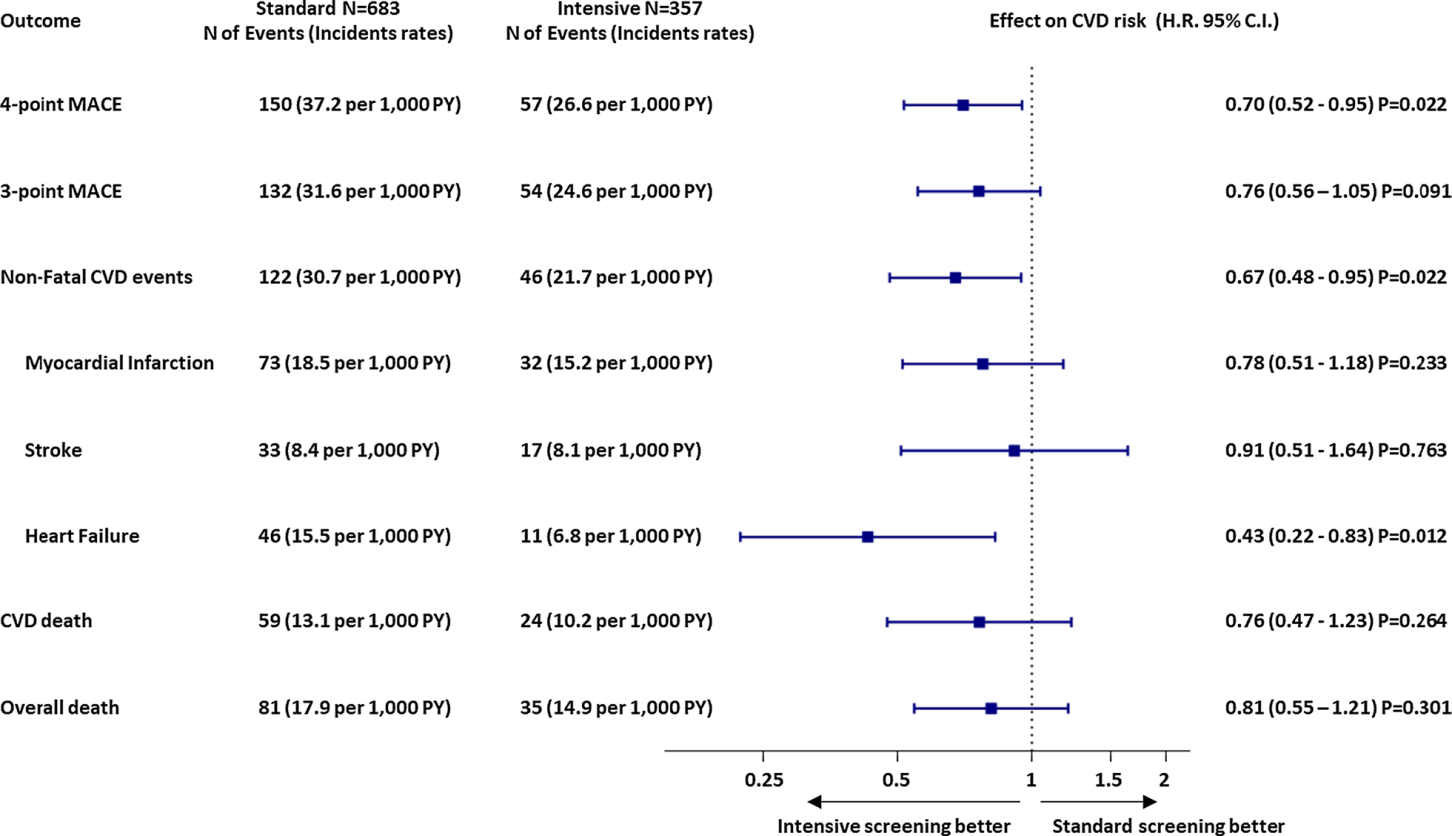
Association between screening program and cardiovascular outcomes. The figure shows the number of each event type and incidence rates (per 1000 PY of follow-up) in the two groups, along with the hazard ratio (with 95% CI) and p values. CVD, cardiovascular disease; MACE, major adverse cardiovascular events; 4-point MACE is defined as cardiovascular mortality, non-fatal myocardial infarction, non-fatal stroke, or hospitalization for heart failure; 3-point MACE is defined as cardiovascular mortality, non-fatal myocardial infarction and non-fatal stroke

During the study, 208 4p-MACE occurred, corresponding to an incidence rate of 34 per 1000 person years. Figure 1 shows the Kaplan–Meier survival curves for 4p-MACE stratified by screening group. After adjusting for baseline prevalence of microangiopathy, the incidence of 4p-MACE was significantly lower in the intensive versus the standard screening group (26.6 vs 37.2/1000 person years; HR 0.70; 95% CI 0.52–0.95; p = 0.022). All components of the primary outcome had nominally lower rates in the intensive versus standard screening group (Fig. 2), which was particularly significant for heart failure (6.8 vs 15.5/1000 person years; HR 0.43; 95% CI 0.22–0.83, p = 0.011). One hundred sixteen subjects died, with 85 deaths (73.3%) being defined as due to cardiovascular causes. Overall mortality rates were similar in the standard and intensive groups (17.9 and 14.9/1000 person years; p = 0.301, respectively) as was the cardiovascular mortality rate (13.1 and 10.2/1000 person years; p = 0.264).

Robustness of these findings was confirmed in the sensitivity analysis conducted in the entire population of 5274 subjects with all baseline information available and analyzed by means of multiple Cox regression models adjusted for all the baseline covariates, yielding results that were equivalent to those obtained on the matched cohorts (Additional file 1: Figure S2).

### Changes in cardiovascular risk factors and medications

**Fig. 3:**
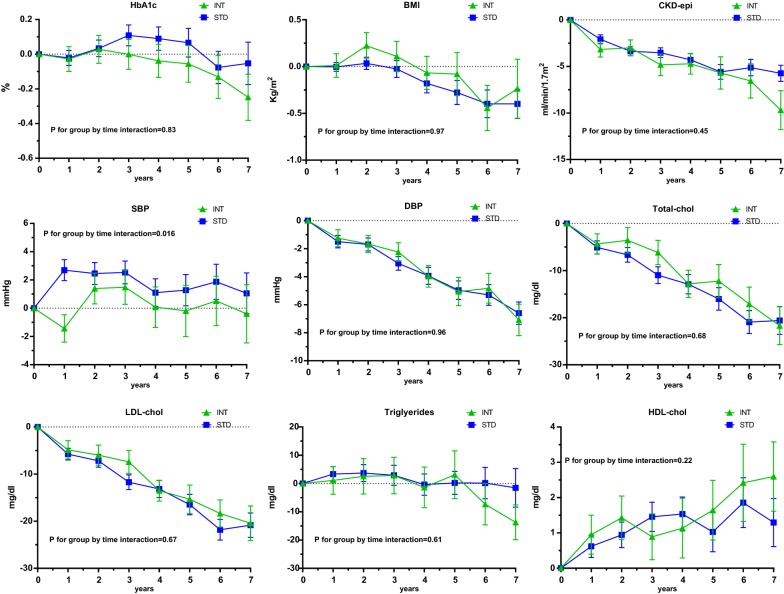
Trends over time of cardiovascular risk factors in the two groups. *INT* intensive screening group, *STD* standard screening group. The x-axis reports time after baseline in years, while the y-axis reports changes in the respective cardiovascular risk factor

Among 1040 patients included in the PSM analysis, 985 (95%) had at least one outpatient visit after the index date at the same Clinic prior to the primary outcome, with the median number of 7 visits (IQR 4 to 13) in both the standard and intensive groups. Few subjects from the intensive and standard groups (29 and 26 respectively) had no information on updated cardiovascular risk factor measures. Figure 3 shows the trends over time of modifiable cardiovascular risk factors in the two groups before 4p-MACE occurrence. Only the trend of systolic blood pressure showed a significant between-group difference, being on average 1.1 mm Hg lower in the intensity group (p for group by time interaction 0.016). The association between intensive complication screening and lower 4-p MACE rates did not change when the changes over time in all cardiovascular risk factor measures were entered in the Cox regression model as time-varying covariates (HR 0.65; 95% CI 0.43–0.98; p = 0.03). We further tested whether the small but significant difference in SBP between the two groups had an influence on the association between intensive screening and lower-HF rates. Additional Cox regression model with SBP changes included as a time-varying covariate yielded unchanged results (HR 0.51; 95% CI 0.26–0.995, p = 0.048), confirming the protective association between intensive complication screening and HF. When the changes in SBP were limited to the first 1.5 years of observation (when the differences in SBP between the two groups were larger), we obtained similar results with or without adjustment for SBP changes. The change over time in the prescription of glucose lowering medications and medications for the control of cardiovascular risk factors showed similar trends in the two groups (Additional file 1: Table S1).

## Discussion

In this study, we show that T2D patients submitted to an intensive and comprehensive 1-day screening of complications had significantly better long-term cardiovascular outcomes than similar T2D patients who underwent routine screening at visits staggered during a year. To the best of our knowledge, this is the first report that the schedule of complication screening can affect cardiovascular outcomes of T2D patients.

Diabetes care in Italy is provided either by specialized diabetes outpatient clinics or by general practitioners. Diabetes clinics are provided with a multidisciplinary team of endocrinologists/diabetologists, trained nurses, podologists and psychologists. Close collaboration with general and vascular surgeons also allows the comprehensive management of the diabetic foot and peripheral arterial disease. Studies performed during the last 10 years reported that patients attending diabetes clinics had lower all-cause mortality rates than those treated by primary care physicians [17]. Though it is impossible to rule out all confounders, those studies highlight the benefits of a comprehensive management of diabetes. Results of our study indicate that, even within the same model of care, the way patients are involved in the screening program is associated with long-term cardiovascular outcome.

Patients receiving screening of micro- and macroangiopathy during a single day session had a 30% lower risk of experiencing a 4p-MACE over the following 6 years than patients whose complications were screened in separate sessions. We calculate that 22 patients (95% CI from 13 to 137) needed to be subjected to intensive screening to prevent one 4p-MACE in the subsequent 5 years. The benefit was particularly evident for the risk of HF, which was reduced by 57%. This result is particularly important since HF is a major cause of hospitalization in diabetic patients in Italy [22]. For this outcome, we estimated that 32 patients (95% CI from 23 to 107) were needed to be exposed to the intensive screening to avoid one hospitalization for HF in the subsequent 5 years. It should be highlighted that, despite in-hospital mortality after a MACE is typically increase in T2D [23], we found no difference between the two groups in the rates of fatal cardiovascular events, and all-cause mortality.

To understand drivers of the different cardiovascular outcome in the two groups, we analyzed the trends in cardiovascular risk factors and medications between baseline and censoring. We found only a nominally significant difference in systolic blood pressure, that may be of limited clinical relevance (1.1 mm Hg) and no significant between-group difference in HbA1c, BMI, eGFR, and lipid profile, nor in the modification of therapies over time. This finding suggests that one or more unmeasured factor(s) was driving the differential outcome between the two groups. We hypothesize that involvement in an intensive screening program improved patients’ awareness and positive attitudes towards diabetes and its complications. The benefit of increasing patients’ awareness have been recently reported in a large population-based cohort study, showing how empowerment programs for patients with T2D was associated with reduction in cardiovascular complications and overall mortality rate [24]. In addition, a complete snapshot of diabetes and complication burden may have driven further diagnostics and appropriate therapeutic tailoring that are not captured by comparing the average change in medications in the two groups. It is possible that, in the intensive screening group more than in the control group, the diagnostic and therapeutic approach was optimized especially in patients at higher cardiovascular risk. The optimized approach might also have led to higher adherence to prescribed treatment in the intensive group (e.g. for instance as suggested by the lower systolic blood pressure despite similar prescription therapy over follow-up) that is known to be associated with reduced risk of hospitalization and all-cause mortality in patients with T2D [25]. HbA1c variability, a well-known proxy of long-term adherence, is in fact associated with cardiovascular outcomes [26]. The intensive screening group, with a more comprehensive approach to the patients, might also have led to a healthier life style (e.g. level of physical activity and dietary pattern) that has been shown to be associated with reduced CV complications independently from established CVD risk factors [27]. However, due to the lack of systematic collection of this information in the EMR, we were unable to evaluate whether changes in lifestyle where influenced by the intensity screening visit.

Interpretation of our findings needs to carefully take into account limitations of this observational study. First, the screening strategy was not randomized, but based on patient’s and diabetologist’s preference. We tried to control for as many confounders as possible: PSM and multiple Cox regression analysis led to the same results, suggesting that known confounders were appropriately handled. It is however impossible to eliminate bias and adjust for unknown confounders due to unmeasured and unmeasurable variables.

## Conclusion

We show that patients subjected to an intensive screening of diabetic complications followed by dedicated counseling had a significantly better cardiovascular outcome than patients subjected to standard staggered complication screening. In the absence of any change in cardiovascular risk factors and medications, such benefit may have resulted from improved patients’ compliance and attitudes towards diabetes management. Due to the possible role of unmeasured confounding factors, the observational design of this study does not allow to draw a firm causal relationship between the single-day intensive screening and the reduced incidence of non-fatal cardiovascular events. However, our results pave the way to the design new studies to validate these findings. Ideally, such studies should be randomized, e.g. by means of the design of a pragmatic clinical trial, in order to circumvent possible residual bias from unmeasured variables that cannot be excluded in observational studies.

## Supplementary information

**Additional file 1.** Additional tables and figures.

## Data Availability

The datasets used and analyzed during the current study are available from the corresponding author on reasonable request.

## Abbreviations

ACE: angiotensin converting enzyme
ARB: angiotensin receptor blockers
BMI: body mass index
CAD: coronary artery disease
CerVD: cerebrovascular disease
CKD: chronic kidney disease
CVD: cardiovascular disease
DPP-4: dipeptidyl peptidase 4
eGFR: estimated glomerular filtration rate
EMR: electronic medical records
GLP-1: glucagon like peptide 1
HDL: high density lipoprotein
HR: hazard ratio
IQR: interquartile range
LDL: low density lipoprotein
MACE: major adverse cardiovascular events
MNSI: Michigan Neuropathy Screening Instrument
PAD: peripheral arterial disease
PSM: propensity score matching
SGLT-2: sodium glucose cotransporter inhibitors
SMD: standardized mean difference
T2D: type 2 diabetes
