# Supplementary material for: Improved long-term cardiovascular outcomes after intensive versus standard screening of diabetic complications: an observational study
Source: Cardiovasc Diabetol. 2019 Sep 16;18:117. doi: 10.1186/s12933-019-0922-1 (PMC6747737; doi:10.1186/s12933-019-0922-1)

**Additional Material**

**Additional file 1: Table S1**. Characteristics of the entire cohorts at the index visit. SMD, standardized mean difference. BMI, body mass index. eGFR, Glomerular Filtration Rate. CKD, Chronic Kidney Disease. ACEi, angiotensin converting enzyme inhibitors. ARBs, angiotensin receptor blockers. CAD coronary artery disease, CerVD cerebrovascular disease, PAD peripheral artery disease.

| **Characteristic** | **Standard screening**  **(N = 4906)** | **Intensive screening**  **(N=368)** | **SMD** | **P** |
| --- | --- | --- | --- | --- |
| **Age** | 69.0 ± 11.6 | 59.7 ± 9.1 | 0.89 | <0.0001 |
| **Female (%)** | 2038 (41.5%) | 121 (32.9%) | 0.18 | 0.001 |
| **Duration of Diabetes (years)** | 10.7 ± 10.1 | 8.3 ± 7.4 | 0.27 | <0.0001 |
| **BMI (kg/m^2^)** | 28.8 ± 4.9 | 29.2 ± 5.0 | -0.09 | 0.106 |
| **Systolic blood pressure (mmHg)** | 139.2 ± 19.9 | 136.4 ± 17.1 | 0.15 | 0.003 |
| **Diastolic blood pressure (mmHg)** | 77.9 ± 10.7 | 81.9 ± 10.1 | -0.38 | <0.0001 |
| **HbA1c (%)** | 7.4 ± 1.3 | 7.6 ± 1.4 | -0.11 | 0.040 |
| **Total Cholesterol (mg/dl)** | 178.5 ± 38.7 | 184.0 ± 36.6 | -0.15 | 0.009 |
| **HDL-chol (mg/dl)** | 51.5 ± 15.2 | 49.1 ± 14.4 | 0.16 | 0.004 |
| **Triglycerides (mg/dl)** | 129.2 ± 72.7 | 135.4 ± 76.7 | -0.08 | 0.118 |
| **LDL cholesterol (mg/dl)** | 101.7 ± 33.2 | 107.8 ± 31.5 | -0.19 | 0.001 |
| **Creatinine (mg/dl)** | 0.99 ± 0.55 | 0.86 ± 0.21 | 0.31 | <0.0001 |
| **eGFR (ml/min/1.7m2)** | 75.4 ± 20.9 | 88.2 ± 17.2 | -0.67 | <0.0001 |
| **Albuminuria** |  |  |  | 0.578 |
| **Normoalbuminuria N (%)** | 3386 (69.0%) | 258 (70.1%) | -0.02 |  |
| **Microalbuminuria N (%)** | 1169 (23.8%) | 89 (24.2%) | -0.01 |  |
| **Macroalbuminuria N (%)** | 351 (7.2%) | 21 (5.7%) | 0.06 |  |
| **Medical Treatment** |  |  |  |  |
| **Any glucose-lowering drugs N (%)** | 4323 (88.1%) | 322 (87.5%) | 0.02 | 0.725 |
| **Metformin alone N (%)** | 1120 (22.8%) | 126 (34.2%) | -0.21 | <0.0001 |
| **Insulin N (%)** | 1483 (30.2%) | 77 (20.9%) | 0.15 | 0.001 |
| **Innovative therapy N (%)** | 535 (10.9%) | 35 (9.5%) | 0.05 | 0.406 |
| **ACEi/ARBs N (%)** | 3254 (66.3%) | 248 (67.4%) | -0.02 | 0.677 |
| **Statin N (%)** | 2707 (55.2%) | 245 (66.6%) | -0.24 | <0.0001 |
| **Lipid-lowering therapy N (%)** | 2901 (59.1%) | 263 (71.5%) | -0.34 | <0.0001 |
| **Anti-platelet therapy N (%)** | 2443 (49.8%) | 114 (31.0%) | 0.40 | <0.0001 |
| **Medical Hystory** |  |  |  |  |
| **CAD or CerVD events N (%)** | 596 (12.1%) | 31 (8.4%) | 0.12 | 0.033 |
| **Macroangiopathy N (%)** | 1834 (37.4%) | 158 (42.9%) | -0.11 | 0.034 |
| **Carotid atheroma or PAD, %** | 31.8% | 41.0% |  |  |
| **CAD, %** | 11.0% | 7.8% |  |  |
| **CerVD, %** | 1.4% | 0.5% |  |  |
| **Microangiopathy N (%)** | 2521 (51.4%) | 210 (57.1%) | -0.11 | 0.036 |
| **Diabetic Nephropathy, %** | 41.9% | 33.1% |  |  |
| **Diabetic Neuropathy, %** | 5.9% | 20.1% |  |  |
| **Diabetic Retinopathy, %** | 16.3% | 24.5% |  |  |
| **CKD N (%)** | 1099 (22.4%) | 31 (8.4%) | 0.39 | <0.0001 |

**Additional file 1: Table S2**. Medications uses during the follow-up in the two groups. Mean (95% C.I. of the mean) For illustration purpose, the difference in the use between the two groups has been described as the average, in each group, of how many time a medication was prescribed in the follow-up visits before MACE-4p occurrence or censoring. ACEi, angiotensin converting enzyme inhibitors. ARBs, angiotensin receptor blockers.

* DPP-4 inhibitors; GLP-1 receptor agonists; SGLT-2 inhibitors.

|  | **Standard** | **Intensive** | **P value** |
| --- | --- | --- | --- |
| **Number of patients** | 654 | 331 |  |
| **No. of visits over follow-up** | 7 (8 - 9) | 7 (8 - 9) | - |
| **Any glucose-lowering drugs** | 90% (88-92) | 92% (89-95) | 0.14 |
| **Metformin alone** | 29% (26-32) | 31% (27-36) | 0.66 |
| **Insulin** | 27% (24-30) | 28% (23-32) | 0.58 |
| **Innovative therapy*** | 22% (19-24) | 21% (17-25) | 0.95 |
| **ACEi/ARBs** | 72% (68-75) | 68% (63-73) | 0.27 |
| **Statin** | 70% (67-73) | 66% (61-70) | 0.30 |
| **Lipid-lowering therapy** | 75% (72-78) | 70% (66-75) | 0.10 |
| **Antiplatelet therapy** | 42% (38-45) | 36% (31-41) | 0.13 |

**Additional file 1: Figure S1**. Study flowchart.

**Additional file 1: Figure S2**. Results of the sensitivity analysis. Multivariable adjusted Cox proportional hazard models were used to calculate hazard ratios. The figure shows the number of each event type and the cumulative percentage in the two groups, along with the hazard ratio (with 95% C.I.) and p values. The model included as covariates all variables used to determine the PS, i.e. all the baseline characteristics described in Table S1, including granularity for glucose lowering medications (diet, metformin alone, or innovative treatment, defined as DPP-4 inhibitors, GLP-1 receptor agonist, or SGLT-2 inhibitors), other medications (ACE inhibitors/angiotensin receptor blockers [ARBs], antiplatelet therapy, statins, or other lipid lowering drugs).


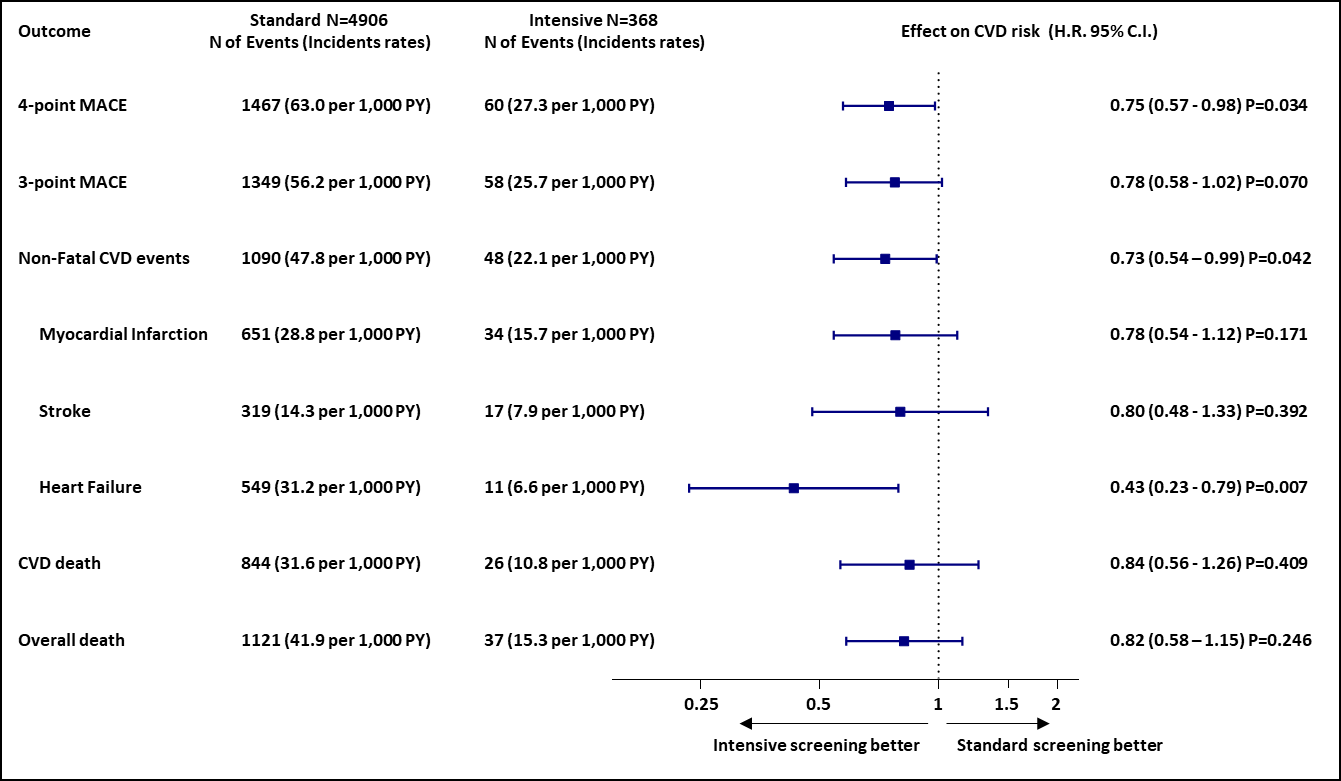

Supplement: Supplementary file 1 — Additional file 1. Additional tables and figures. [file 12933_2019_922_MOESM1_ESM.docx]
